# Whole-Body Photoacoustic Imaging Techniques for Preclinical Small Animal Studies

**DOI:** 10.3390/s22145130

**Published:** 2022-07-08

**Authors:** Hyunjun Kye, Yuon Song, Tsedendamba Ninjbadgar, Chulhong Kim, Jeesu Kim

**Affiliations:** 1 Departments of Cogno-Mechatronics Engineering and Optics & Mechatronics Engineering, Pusan National University, Busan 46241, Korea; hyunjuijui@pusan.ac.kr (H.K.); dhflakdl@pusan.ac.kr (Y.S.); ninjee97@pusan.ac.kr (T.N.); 2 Departments of Convergence IT Engineering, Mechanical Engineering, and Electrical Engineering, School of Interdisciplinary Bioscience and Bioengineering, Medical Device Innovation Center, Pohang University of Science and Technology (POSTECH), Pohang 37673, Korea

**Keywords:** photoacoustic imaging, whole-body imaging, small animal imaging, biomedical imaging

## Abstract

Photoacoustic imaging is a hybrid imaging technique that has received considerable attention in biomedical studies. In contrast to pure optical imaging techniques, photoacoustic imaging enables the visualization of optical absorption properties at deeper imaging depths. In preclinical small animal studies, photoacoustic imaging is widely used to visualize biodistribution at the molecular level. Monitoring the whole-body distribution of chromophores in small animals is a key method used in preclinical research, including drug-delivery monitoring, treatment assessment, contrast-enhanced tumor imaging, and gastrointestinal tracking. In this review, photoacoustic systems for the whole-body imaging of small animals are explored and summarized. The configurations of the systems vary with the scanning methods and geometries of the ultrasound transducers. The future direction of research is also discussed with regard to achieving a deeper imaging depth and faster imaging speed, which are the main factors that an imaging system should realize to broaden its application in biomedical studies.

## 1. Introduction

Photoacoustic imaging (PAI) is a non-invasive biomedical imaging technique based on the photoacoustic (PA) effect that involves energy transduction from light to sound [1]. In recent decades, PAI has gained considerable attention in biomedical research owing to its unique characteristics [2,3,4]. PAI is cost efficient and easy to implement compared to other medical imaging techniques, such as X-ray imaging, X-ray computed tomography, magnetic resonance imaging, and positron emission tomography. In addition, PAI is free from ionizing radiation, which may cause side effects in biological tissues. Similar to optical imaging techniques, PAI can provide molecular functional information using multispectral data acquisition [5,6,7,8,9], which is not available in ultrasound imaging (USI). By contrast, PAI can deeply penetrate biological tissue, similar to USI, whereas the typical imaging depth of pure optical imaging is ~1 mm (i.e., optical mean free path) [10,11].

The PA effect occurs when a short (~10 ns) pulsed laser is absorbed by chromophores in biological tissue. The absorbed light energy is released as thermal energy, and thermoelastic expansion causes a volumetric change in the surrounding tissues. Because thermal energy is rapidly dissipated owing to the short pulse width of the excitation laser, the expanded tissues shrink to their original size. The repeated volume changes generate vibrations that propagate in the form of acoustic waves called PA waves. By detecting these acoustic waves using US transducers, PA images can be obtained through an image generation procedure, which is similar to that of US image generation.

PAI has another unique characteristic: scalable resolution and imaging depth according to the target [12]. Based on the principles of PAI, the resolution and imaging depth can be controlled by adjusting the light illumination method and transducer geometry. When light is tightly focused, high-resolution (~5–50 μm) photoacoustic microscopy (PAM) can be implemented [13,14,15]. However, PAM is limited in imaging depth (~1 mm); thus, it is primarily applied to imaging superficial areas, including the ear, eye, brain, and skin in small animals [16,17,18,19,20,21]. The imaging depth of PAI can be enhanced (up to ~10–20 mm) by sacrificing its spatial resolution (~100–500 μm) [22]. In such configurations, the light is moderately focused or even diffused in biological tissue, and the resolution of images is determined by the acoustic focal zone of the US transducers.

This review summarizes the configurations of PAI systems used to achieve deep-tissue images in large regions, particularly for whole-body imaging of small animals in vivo. Various US transducer apparatuses (single-element and array transducers) and their scanning mechanisms used to obtain PA images are explored. The imaging performances are then compared, focusing on the imaging area, spatial resolution, and imaging speed. This review will assist with the implementation of PAI systems for preclinical small animal studies, which require a wide imaging area and include drug-delivery monitoring, visualization of biodistribution, agent tracking, and treatment evaluation.

## 2. Photoacoustic Imaging with Single-Element Transducer

**Figure 1:**
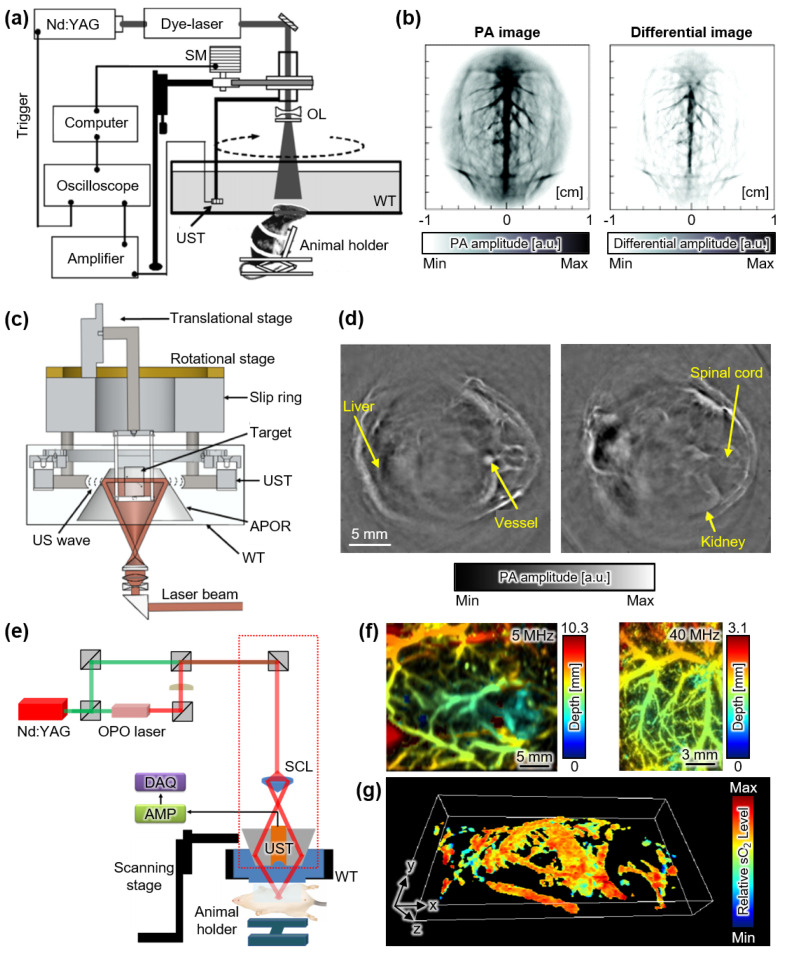
PAI systems with single-element USTs. (**a**) Schematic of PAI system with circular scanning of a single-element UST. (**b**) Contrast-enhanced PA and differential images of the brain of a rat with an injection of contrast agent. (**c**) Schematic of PAI system with a slip-ring-based rotational scanning using multiple USTs. (**d**) PA images of abdomen region of mice in vivo. (**e**) Schematic of PAI system with raster scanning using a single-element UST. (**f**) PA images with two different UST center frequencies. (**g**) Volumetric SaO_2_ whole-body distribution in mice in vivo. PA, photoacoustic; PAI, photoacoustic imaging; UST, ultrasound transducer; SaO_2_, hemoglobin oxygen saturation; SM, step motor; OL, optical lens; WT, water tank; APOR, acoustically penetrable optical reflector; AMP, amplifier; DAQ, data acquisition module; SCL, spherical conical lens. The images are reproduced with permission from refs. [23,25,26,27].

Initially, PAI systems used single-element US transducers to acquire data. Single-element transducers scanned around the animals to produce tomographic images. Wang et al. demonstrated a tomography device with cross-sectional brain images of rats using a Nd:YAG pulsed dye laser [23]. In the system, an unfocused US transducer with a center frequency of 10.4 MHz was submerged in a water tank to detect PA waves (Figure 1a). A circular rotational scan using a US transducer with a step angle of 1.5° and scanning radius of 3 cm was performed around a rat’s head to acquire cross-sectional PA images of the brain (Figure 1b). To increase contrast, light-absorbing metal nanoshells were injected into the rat intravenously. The resulting images visualized the blood vessels in the brain with a spatial resolution of ~60 μm and an imaging depth of ~1 cm. Differential images before and after the injection of the contrast agent delineated the blood vessels in the brain. However, the imaging time required for a single cross-sectional image was slow (~24 min) owing to the mechanical scanning of the transducer using a stepping motor.

Ma et al. developed a multispectral PAI system to improve the imaging speed using a Nd:YAG pulsed optical parametric oscillator (OPO) laser [24]. The system was equipped with a single-element focused US transducer with a center frequency of 3.5 MHz. Interestingly, a rotational stage was used to move the imaging targets instead of scanning the US transducer. The thoracic and neck regions of mice were successfully visualized with a spatial resolution of 32 μm and depth of ~7 mm. The imaging speed required for a single cross-sectional image was reduced to 9 s owing to the faster rotational scan with an angular speed of 40°/s, which corresponded to 6.7 rotations per minute.

Deng et al. also attempted to improve the imaging speed using a slip-ring-based PAI system with a Ti:Sapphire laser [25]. They used two single-element focused US transducers with a center frequency of 5 MHz. The two transducers were symmetrically positioned in the slip-ring-based scanning stage; thus, full data for the cross-sectional image could be obtained with only half a rotation (Figure 1c). Tomographic images of the abdominal regions of mice were acquired. The resulting images had a spatial resolution of ~129 μm with a total imaging time of 9 min (Figure 1d).

For a simple configuration, Jeon et al. used raster scanning with a single-element focused transducer to achieve whole-body images of mice in vivo [26]. The system could switch the US transducers between two different center frequencies of 5 and 40 MHz (Figure 1e). Using raster scanning, the system obtained landscape images by projecting the most significant signal in the volumetric data to the transverse plane (Figure 1f). The two different center frequencies of the transducers produced different imaging performances: high spatial resolution (~85 μm) in the shallow region (~3.1 mm) and a relatively low spatial resolution (~590 μm) in the deeper region (~10.3 mm). However, the imaging speed of this system was limited (~20 min for a scanning region of 60 × 32 mm^2^) owing to the slow mechanical scanning of the transducer. Although volumetric data were obtained in this system, three-dimensional (3D) visualization was limited owing to breath-related distortion during the long scanning time. Recently, Lee et al. improved the system to achieve both US and PA images simultaneously and produced breath-compensated 3D PA whole-body images of mice [27,28]. Breath-related distortion was corrected by segmenting the skin profile in US images, realigning the signals in the axial direction, and applying the realignment parameters to the corresponding PA data. Breath compensation was also applied to the multispectral data to generate 3D hemoglobin oxygen saturation whole-body maps of mice (Figure 1g).

## 3. Photoacoustic Imaging with Array Transducer

Multi-element array transducers are widely used for PAI to improve imaging speed. Various transducer element geometries can be implemented for PAI, including linear, curved, and bowl-shaped geometries. The imaging speed can be significantly improved in array-based PAI systems compared to single-element PAI systems because multiple elements achieve PA signals simultaneously, thereby producing a larger imaging region with a single laser pulse. The spatial resolution and image quality may be degraded by mathematical image reconstruction procedures; however, the fast imaging speed and deep penetration depth of array-based PAI systems are beneficial for biomedical applications, especially in clinical human research [29,30,31,32]. 

### 3.1. Linear Array Transducers

**Figure 2:**
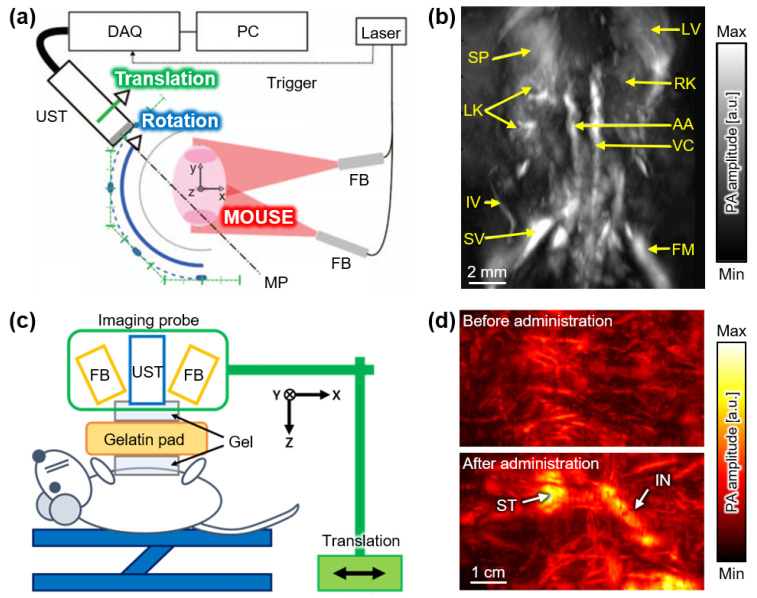
PAI systems with linear array USTs. (**a**) Schematic of PAI system with a combination of translational and rotational scans using a linear array UST. (**b**) Representative PA image of abdomen area of a mouse in vivo. (**c**) Schematic of PAI system with a simple translational scan using a linear array UST. (**d**) PA images of abdomen area of a rat in vivo before and after oral administration of exogenous contrast agents. PA, photoacoustic; PAI, photoacoustic imaging; UST, ultrasound transducer; FB, fiber bundle; DAQ, data acquisition module; SP, spleen; LV, liver; LK, left kidney; RK, right kidney; AA, abdominal aorta; VC, vena cava; IV, ischiatic vein; SV, saphenous vein; FM, femur; ST, stomach; IN, intestine. The images are reproduced with permission from refs. [33,39].

Gateau et al. demonstrated a PAI system for obtaining tomographic images of mice ex vivo using a linear array transducer, which consisted of 128 elements with a center frequency of 7 MHz [33]. Whole-body volumetric PA images were obtained by moving the linear array transducer using a combination of translational and rotational scans (Figure 2a). A rotational scan was performed with an angular step of 1.5° for a total of 180° (blue path in Figure 2a). At each rotational point, a translational scan was performed with a step size of 1.5 mm for a total length of 13.5 mm (green path in Figure 2a). The total number of data acquisition points was 1140, and it took ~1.5 h to perform the entire scan. The abdominal area of a 7-day-old mouse was imaged using the combined scanning method (Figure 2b). Internal organs and major vessels were successfully visualized with a lateral resolution of 130 μm, an elevational resolution of 330 μm, and an imaging depth of ~7.5 mm. Although 3D PA images with adequate quality were generated in this study, the scanning mechanism was complicated and slow for in vivo applications. 

Needles et al. developed a PAI system with a high-frequency transducer array, which was the initial form of the Vevo LAZR system (VisualSonics, Toronto, Canada) [34]. The system used a 256-element linear array with a center frequency of 21 MHz. Three-dimensional PA images of the abdominal region of a mouse were acquired using simple translational scanning in the elevational direction. The high-frequency transducer visualized the superficial blood vessel network well; however, the imaging depth was limited to ~5 mm. Despite the shallow imaging depth, the laser source (Nd:YAG pumped OPO laser) enabled multispectral analyses of small animals, especially the visualization of hemoglobin oxygen saturation (SaO_2_) levels with high resolution. Therefore, this system and its subsequent forms have been widely used in small animal studies [35,36,37,38]. 

To achieve deeper imaging, Kim et al. demonstrated a PAI system consisting of an Nd:YAG pumped OPO laser and a 128-element 8.5 MHz linear array transducer [39]. Similar to the Vevo LAZR system, a simple translation was performed to obtain volumetric data of the abdominal region of rats (Figure 2c). In this study, translational scanning was performed using a manual translational stage. In addition to the successful visualization of blood vessels and internal organs with a lateral resolution of 1.2 mm and imaging depth of ~1 cm, contrast-enhanced PA images of the gastrointestinal tract were acquired after oral administration of exogenous agents (Figure 2d). Using the same system, Park et al. demonstrated deep-tissue imaging using contrast agents with strong optical absorption in the second near-infrared (NIR-II) region [40,41]. The scanning speed was significantly increased (less than 30 s) using a motorized translational stage. The imaging region was scanned at a speed of 2 mm/s, which corresponded to a step size of 0.2 mm. The imaging depth was improved to ~5 cm owing to the use of optical absorbing agents. In addition to its deep imaging capability, the system utilized an FDA-cleared US machine; thus, it is promising for clinical translation [42]. 

### 3.2. Curved Array Transducers

**Figure 3:**
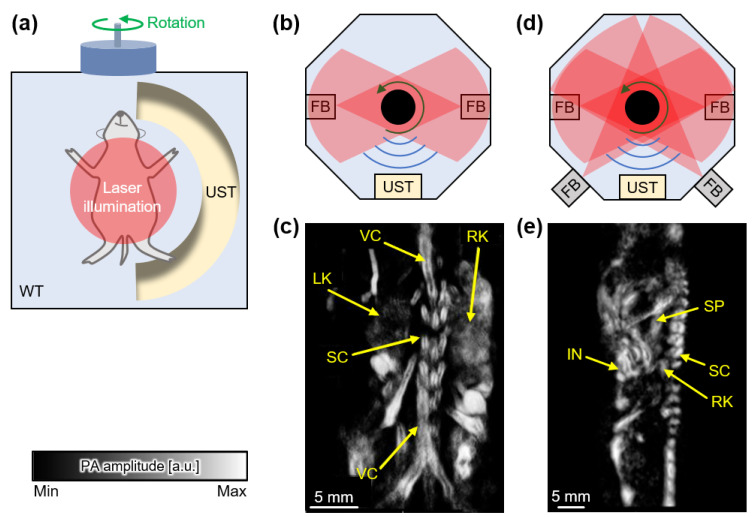
PAI systems with curved array USTs. (**a**) Schematic of PAI system with a rotational scan using a curved array UST. (**b**) Schematic of light illumination and PA wave acquisition in the initial configuration. (**c**) Volumetric PA whole-body image of mice in vivo. (**d**) Schematic of light illumination and PA wave acquisition in the updated system. (**e**) PA whole-body images of a mouse in vivo with the additional light illuminating FBs. PA, photoacoustic; PAI, photoacoustic imaging; UST, ultrasound transducer; FB, fiber bundle; VC, vena cava; LK, left kidney; RK, right kidney; SC, spinal cord; SP, spleen; IN, intestine. The images are reproduced with permission from refs. [44,46].

Curved array transducers have been widely developed and applied in small animal studies to acquire tomographic PA images [43]. At the initial stage, arc-shaped array transducers with rotational scanning were implemented in PAI systems. Brecht et al. demonstrated whole-body PAI of mice in vivo with a concave, 64-element, arc-shaped array transducer which had a center frequency of 3.1 MHz, focal length of 65 mm, and angular aperture of 152° (Figure 3a) [44]. The curved array transducer was rotated around the mice with an angular step of 2.4° for a full 360° scan range, which required 8 min to achieve full data (Figure 3b). The resulting whole-body images of the mice exhibited internal organs and major blood vessels with a spatial resolution of ~500 μm (Figure 3c). The system was equipped with two lasers for multi-wavelength excitation (Alexandrite laser at 755 nm and Nd:YAG laser at 1064 nm); however, multispectral analysis was not performed in this study. Wang et al. also demonstrated an iterative image reconstruction process for the same system to suppress background artifacts while preserving spatial resolution [45]. Su et al. improved the PAI system to achieve better image quality [46]. In the updated system, the laser beams were transmitted through four fiber bundles so that uniform light illumination was applied to the target objects (Figure 3d). Volumetric PA images were acquired using full circular scanning with an angular step of 2.4°. To evaluate the system, they assessed the clearance of exogenous dye by monitoring biodistributions 24–48 h post injection (Figure 3e). 

Razansky et al. reported an arc-array-based PAI system with a different scanning mechanism [47]. For 3D whole-body imaging, the mice were moved perpendicular to a curved array transducer comprising 64 transducer elements with a center frequency of 5 MHz and an angular aperture of 172°. The laser was used to scan around the mice to produce PA signals across the entire bodies of the mice. Cross-sectional images were acquired at a transverse resolution of 150 μm. The mice were scanned using a linear translation stage with a step size of 0.4 mm and a scanning range of 150 mm, and 3D whole-body images were acquired in 15–30 min. For multispectral imaging, the system was equipped with a tunable OPO laser pumped by a Nd:YAG laser. Using the multispectral data, unmixed signals were successfully overlaid on the conventional PA images, thereby exhibiting the biodistribution of the administered exogenous agent.

**Figure 4:**
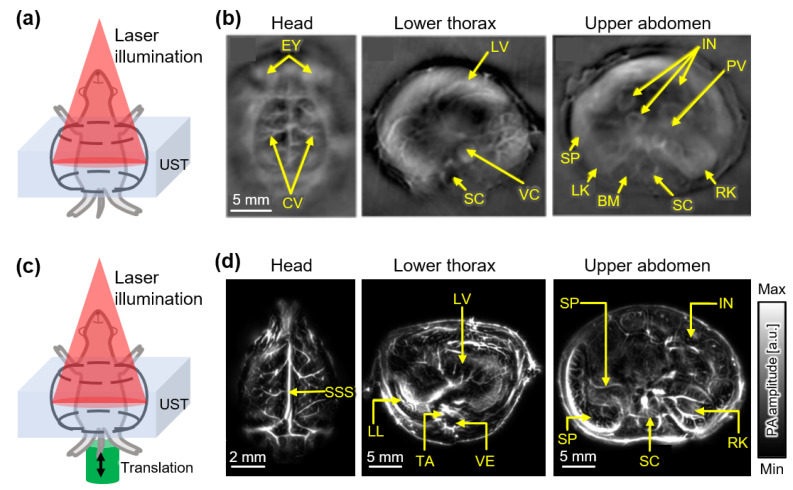
PAI systems with full-ring array USTs. (**a**) Schematic of PAI system with a curved array UST. (**b**) Cross-sectional PA images at the various positions of a mouse in vivo. (**c**) Schematic of PAI with a translational scan of mice for volumetric data acquisition. (**d**) Cross-sectional PA images from the volumetric whole-body image of a mouse in vivo. PA, photoacoustic; PAI, photoacoustic imaging; UST, ultrasound transducer; EY, eyes; CV, cortical vessels; LV, liver; SC, spinal cord; VC, vena cava; SP, spleen; LK, left kidney; RK, right kidney; BM, backbone muscle; PV, portal vein; IN, intestine; SSS, superior sagittal sinus; LL, left lung; TA, thoracic aorta; VE, vertebra. The images are reproduced with permission from refs. [48,49].

Full-ring arrays have also been used to obtain tomographic images to cover all the PA waves generated from small animals. Xia et al. demonstrated a full-ring array transducer to acquire cross-sectional images of mice in vivo (Figure 4a) [48]. In the full-ring array transducer, 512 cylindrically focused elements with a center frequency of 5 MHz were evenly distributed with a ring diameter of 50 mm. A tunable Ti:Sapphire laser beam was deformed into a ring shape using optical components and transmitted to the imaging plane. A core imaging region 20 mm in diameter and 1 mm in thickness was generated by the combined foci of all transducer elements. The resulting images successfully visualized the internal anatomies of mice with a transversal resolution of ~100 μm and imaging speed of 1.6 s (Figure 4b). Li et al. added a motorized translational stage to the imaging target to obtain 3D images with a faster imaging speed [49]. By scanning the full-ring array transducer in the elevation direction, 3D PA images were obtained with a transverse resolution of 125 μm. The total imaging time was 12 s, and the images included 600 cross-sectional positions. The full anatomies of mice were successfully visualized using an Nd:YAG laser with a wavelength of 1064 nm and a repetition rate of 50 Hz (Figure 4c). The internal organs of mice, including the heart, lung, liver, spleen, kidney, and intestines, as well as the vasculature of the brain cortex in rats, were imaged. In addition, PA images of human breasts were visualized using the same system. Thus, the system exhibited considerable potential for use in clinical applications, particularly in breast-related studies [50].

### 3.3. Photoacoustic Imaging with Spherical Array

**Figure 5:**
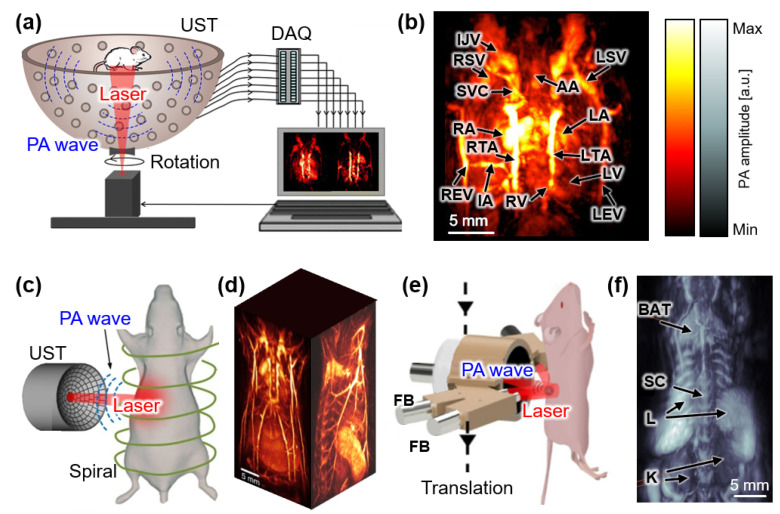
PAI systems with spherical array USTs. (**a**) Schematic of PAI system with a hemispherical array UST. (**b**) Volumetric PA image of the heat in a mouse. (**c**) Schematic of PAI system with a spiral scan using a spherical array UST. (**d**) Volumetric visualization of whole-body images of a mouse in vivo. (**e**) Schematic of PAI system with a translation scan using a spherical array UST. (**f**) Volumetric PA whole-body image of a mouse in vivo. PA, photoacoustic; PAI, photoacoustic imaging; UST, ultrasound transducer; DAQ, data acquisition module; FB, fiber bundle; IJV, internal jugular vein; RSV, right subclavian vein; LSV, left subclavian vein; SVC, superior vena cava; AA, aortic arch; RA, right atrium; LA, left atrium; RTA, right thoracic artery; LTA, left thoracic artery; RV, right ventricle; LV, left ventricle; IA, intercostal artery; REV, right epigastric vein; LEV; left epigastric vein; BAT, brown adipose tissue; SC, spinal cord; L, liver; K, kidney. The images are reproduced with permission from Refs. [52,55,56].

In PAI, PA waves propagate in all directions in the surrounding tissue from the origin of the optical absorption. Spherical array transducers have been developed and applied to PAI systems to cover these omnidirectional PA waves [43,51]. Lv et al. demonstrated a PAI system equipped with a 128-element hemispherical transducer with a center frequency of 5 MHz [52]. A circular scan with a total time of ~1.7 min was performed to obtain volumetric images of mice in vivo (Figure 5a). The acquired 3D images of mouse hearts clearly indicated the position of blood vessels with a spatial resolution of ~200 μm and an imaging depth of ~10 mm (Figure 5b). The system was also evaluated via the monitoring of myocardial infarction using PA images over 11 days.

Deán-Ben et al. also demonstrated a spherical array transducer for PAI [53]. The transducer consisted of 256 piezoelectric elements placed on a partially spherical surface with a covering angle of 90°. The individual size of each piezoelectric element was 3 × 3 mm^2^, and they had a center frequency of 4 MHz. The feasibility of the transducer was tested using cerebral vascular images of mice in vivo after they were injected with a contrast agent [54]. Multispectral data were acquired at five different wavelengths over a total imaging time of 100 ms. The resulting images displayed the flow of the injected agent in a volumetric field of view of 10 × 10 × 15 mm^3^. The imaging area was expanded to the entire body of mice by adding a spiral scanning mechanism (Figure 5c) [55]. The total scanning time for the spiral trajectory around the mice increased to ~5 min, and the resulting 3D images were successfully acquired with a spatial resolution of 200 μm (Figure 5d). Recently, the authors reduced the imaging speed to 1.8 s while maintaining the spatial resolution [56]. In the proposed system, volumetric PA images were acquired via single-sweep scanning using the spherical array transducer (Figure 5e). The key difference compared with their previous system was the number of laser illuminations. They used multi-beam illumination to cover a larger area, thereby producing PA waves across the entire body of the mouse. The resulting images exhibited the considerable potential of this approach to provide high-speed, whole-body images of mice in vivo (Figure 5f). 

Recently, Lin et al. also developed a high-speed 3D PAI system using four integrated 256-element curved array modules with a center frequency of 2.25 MHz [57]. The integrated arrays obtained data similar to those of hemispherical arrays when rotational scanning was performed. A total of 1064 elements were connected to the 1064 channels; therefore, no multiplexing was required, which resulted in a fast imaging speed (5 s for the entire scan). Using this system, volumetric images of the mouse brain were acquired with a spatial resolution of ~390 μm and an imaging depth of 10 mm. The fast imaging speed enabled functional analyses of the brain. In addition, human female breasts were imaged using the same system configuration, thereby demonstrating this technique’s potential for use in clinical applications.

## 4. Conclusions and Outlook

PAI is a promising biomedical imaging technique that can be used to assess biodistribution in small animals in vivo. By detecting the optical absorption characteristics of biological tissue with US resolution, PAI can visualize molecular functional information in deep tissue better than pure optical imaging techniques. Whole-body visualization of small animals is widely applied in preclinical biomedical studies, including drug-delivery monitoring [58], treatment assessment [59], and contrast-enhanced imaging [60]. For these purposes, various configurations of PAI systems have been demonstrated using various combinations of US transducers and scanning mechanisms. 

In this review, we discussed PAI systems used for whole-body imaging of small animals with respect to the geometries of US transducers, i.e., single-element, linear array, curved array, and spherical array transducers (Table 1). Depending on the transducer type, appropriate scanning mechanisms (typically linear translational scanning or rotational scanning) were used to obtain volumetric images of small animals in vivo.

Single-element transducers can achieve higher resolution compared to array transducers by tightening either the optical or acoustic focal zone. In single-element PA systems, an individual A-line signal (time-resolved signals along the depth direction) is acquired at each position; thus, PA images can be reconstructed without complex mathematical algorithms. However, they typically require a long scanning time for volumetric data acquisition, which limits temporal monitoring of the whole-body distribution of small animals in vivo. Array-based systems can overcome the slow imaging speed by acquiring data for multiple elements with a single laser illumination. Although they may suffer from mathematical artifacts generated by image reconstruction algorithms, volumetric images with a larger field of view can be achieved with a much faster data acquisition time compared to single-element systems. According to the geometry of the array, adequate scanning mechanisms are applied. Linear array transducers are typically utilized for investigating biodistribution in cross-sectional, B-mode images. They can achieve volumetric data by translational scanning in the elevational direction. Although the spatial resolution is not good compared to the curved and spherical array transducers, linear array transducers have great potential to expand the application area from preclinical small animals to clinical human studies based on their hand-held operation capability. Curved array transducers acquire volumetric data by rotational scanning of the arc-shaped array or translational scanning of the ring-shaped array. They achieve good image quality in small animals, but the mechanical scanning around the animals requires a relatively long scanning time and complex system configuration. Spherical array transducers can achieve volumetric data with single laser illumination; thus, the image acquisition speed is faster than in other types of transducer. They can extend their volumetric field of view by using various scanning methods, including rotational, helical, and translational scanning. Bowl-shaped geometry can also be applied for human applications, especially for breast imaging. 

In addition to small animal studies, the principles of PAI techniques using array transducers can be directly applied to clinical human studies by expanding the scanning range. In recent years, a variety of clinical trials have been conducted, particularly for visualizing tumors, including breast [61,62,63,64,65,66,67,68], thyroid [69,70,71], melanoma [72,73,74], and prostate [75,76] cancers. For successful clinical translation, several improvements should be made to existing PAI systems. First of all, fast imaging speed is required to minimize distortions from patient movement. For this purpose, parallel processing algorithms for signal processing and image reconstruction have been investigated in clinical systems [77,78]. However, the current bottleneck for imaging speed is mainly due to the pulse repetition rate of laser sources. Therefore, the development of faster laser sources could significantly improve the imaging speed [79]. Secondly, the light-delivering mechanism should be optimized according to the application area, because the initial pressure of PA waves is linearly proportional to the optical fluence. Monte Carlo simulations have been used for investigating optimal light delivery [80,81,82], and several studies were conducted for designing integrated PA and US probes [83,84]. The fundamental limitation of the current probe is the integration of the separated US transducer and light-delivery system. Developing an integrated probe that consists of optical fibers inside of the US transducer can significantly improve the light delivery efficiency. Transparent US transducers, which have recently been introduced, are another option for improving light delivery efficacy [85,86]. In addition, adequate image reconstruction algorithms with volumetric evaluation can improve the visibility of the resulting images [87,88,89]. In addition to traditional delay-and-sum or model-based algorithms, deep learning techniques have been widely investigated in recent years [90]. Deep learning methods have been variously applied in PAI, including in image reconstruction with improved resolution [91,92] or signal-to-noise ratio [93], quantitative image acquisition [94], correction of the speed of sound [95], and image segmentation [96]. These enhancements can also be applied to preclinical small animal imaging for better spatiotemporal resolution, which will expand the application area of PAI systems.

## Figures and Tables

**Table 1:** The characteristics of USTs, scanning mechanisms, and resulting images in PAI systems. UST, ultrasound transducer; PAI, photoacoustic imaging; *f_c_*, center frequency; *N_e_*, number of elements; FOV, field of view; WB, whole-body.

UST			Scanning			Imaging			
Type	*f_c_* [MHz]	*N_e_*	Type	FOV [mm^2^]/[mm^3^]	Time	Depth [mm]	Resolution [μm]	Application	Ref.
Single- element	10.4	1	Rotation (UST)	20 × 20	~24 min	~10	~60	Brain (rat)	[23]
	3.5	1	Rotation (Animal)	25 × 25	~9 s	~7	~32	Thorax, neck (mouse)	[24]
	5	1	Rotation (UST)	20 × 20 × 10	9 min	~9.5	~129	WB (mouse)	[25]
	5	1	Raster (UST)	60 × 32	~20 min	~10.3	~590	WB (mouse)	[26]
Linear array	7.0	128	Rotation + Translation (UST)	13.5 × 13.5 × 160	1.5 h	~13.5	~130	WB (mouse)	[33]
	21	256	Translation (UST)	20 × 35 × 8	N/A	~4	N/A	WB (mouse)	[34]
	8.5	128	Translation (UST)	40 × 75 × 45	30 s	~46	~1200	WB (rat)	[39]
Curved array	3.1	64	Rotation (UST)	40 × 40 × 60	~8 min	~65	~500	WB (mouse)	[44]
	3.1	64	Rotation (UST)	25 × 25 × 70	N/A	~65	N/A	WB (mouse)	[46]
	5	64	Rotation (UST)	25 × 25 × 150	~30 min	~50	~150	WB (mouse)	[47]
	5	512	Translation (Animal)	25 × 25 × 40	~25 min	~19	~100	WB (mouse)	[48]
	5	512	Translation (Animal)	30 × 30 × 35	~12 s	~11	~125	WB (mouse)	[49]
Spherical array	5	128	Rotation (UST)	21 × 13 × 28	~1.7 min	~10	~200	Heart (mouse)	[52]
	4	256	Helix (UST)	21 × 21 × 42	~5 min	~10	~250	WB (mouse)	[55]
	7	512	Translation (UST)	22 × 21 × 57	~6.9 s	~10	~200	WB (mouse)	[56]
	2.25	1064	Rotation (UST)	16 × 10 × 21	~5 s	~10	~390	Brain (rat)	[57]

## Data Availability

Not applicable.
